# Editorial: Directing Stem Cell Fate Using Plant Extracts and Their Bioactive Compounds

**DOI:** 10.3389/fcell.2022.957601

**Published:** 2022-06-29

**Authors:** Farhana Ferdousi, Kazunori Sasaki, Dongzhu Xu, Yun-Wen Zheng, Francis G Szele, Hiroko Isoda

**Affiliations:** ^1^ AIST-University of Tsukuba Open Innovation Laboratory for Food and Medicinal Resource Engineering (FoodMed-OIL), AIST, University of Tsukuba, Tsukuba, Japan; ^2^ Alliance for Research on the Mediterranean and North Africa (ARENA), University of Tsukuba, Tsukuba, Japan; ^3^ Cardiovascular Division, Institute of Clinical Medicine, Faculty of Medicine, University of Tsukuba, Tsukuba, Japan; ^4^ Department of Gastrointestinal and Hepato-Biliary-Pancreatic Surgery, Faculty of Medicine, University of Tsukuba, Tsukuba, Japan; ^5^ Department of Physiology, Anatomy and Genetics, University of Oxford, Oxford, United Kingdom; ^6^ Faculty of Life and Environmental Sciences, University of Tsukuba, Tsukuba, Japan

**Keywords:** plant extract, bioactive compound, stem cell differentiation, neurogenesis, mesenchymal stem cell, human amniotic epithelial cells, hair growth, anticancer activity

The rapidly evolving field of stem cell therapy has great potential for treating a broad array of diseases, including currently incurable diseases. However, the use of stem cell-based products as therapeutics is often limited by high rejection rate, insufficient availability, and expensive *in vitro* expansion methods (McNeish, 2004; Laustriat et al., 2010; Rubin and Haston, 2011). The external control of stem cells using plant-derived bioactive compounds, such as polyphenols, flavonoids, tannins, terpenoids, and fatty acids, may provide potential solutions to overcome many of these current limitations. Many naturally occurring bioactive compounds have been shown to promote stem cell proliferation and lineage-specific differentiation (Udalamaththa et al., 2016). This Research Topic aimed to cover promising and novel research findings on the effects of plant extracts and their bioactive compounds on regulating cell division and differentiation of pluripotent and adult stem cells and stem cells obtained from alternative sources. This article Research Topic includes ten original research articles, one brief research report, and one review article covering the potentials of bioactive compounds for neurodegenerative, cardiovascular, metabolic, cancer, musculoskeletal, and hair loss diseases through regulating stem cell proliferation and differentiation. Below we present the focus and key findings of each article.

Mesenchymal stem cells (MSCs) are multipotent progenitor cells that can be differentiated into skin cells, such as fibroblasts and keratinocytes. Apart from their differentiation capacity, MSCs exert unique paracrine actions to accelerate wound healing and maintain tissue homeostasis and, therefore, have been regarded as a potentially promising therapeutic option for tissue injury and diseases (Guillamat-Prats, 2021). Plant-derived components that can promote biological events, such as migration and homing of MSCs, may offer novel therapeutic options for regenerative medicine. The review by Maeda addresses the role of plant-derived components in promoting the migration and homing of MSCs to damaged sites, where they contribute to the healing process (Maeda).

The following group of five original research articles was dedicated to exploring the potential of plant components on neurodegenerative and neuropsychiatric conditions through regulating neural stem cells (NSCs) proliferation and differentiation *in vitro* and *in vivo* (Houghton et al.; Iwata et al.; Achour et al.; He Y. et al.; Sasaki et al.).

An interesting paper by Houghton et al. reported that exposure to supraphysiological caffeine condition could significantly reduce progenitor integrity and proliferation of human hippocampal progenitor cells, HPC0A07/03, compared to control conditions. This finding indicates that regular dietary components, such as caffeine, can affect cognitive outcomes by influencing NSC integrity and proliferation and highlights the potential of leveraging dietary interventions to promote cognitive health.

Iwata et al. evaluated the benefits of sugarcane (Saccharum officinarum L.) top as a putative dietary supplement to improve aging consequences using *in vitro* and *in vivo* strategies. They showed that the ethanolic extract of sugarcane top (STEE), rich in caffeoylquinic acid derivatives, could rescue age-associated decline in spatial learning and memory, increase the number of newborn neurons in the subgranular zone of hippocampus, and restore the levels of neurotransmitters in the cerebral cortex of senescence-accelerated mouse SAMP8. They also demonstrated that STEE enhanced cellular energy metabolism through upregulation of glycolytic reaction in human neuroblastoma cells, SH-SY5Y, and positively affected proliferation, induced neuronal differentiation and astrocyte morphogenesis in human NSCs (hNSCs).

He Y. et al. analyzed the effect of *Alpinia oxyphylla* Miq. extract (AOM) and its bioactive compound *p*-coumaric acid (*p*-CA) on post-stroke recovery in rats. They reported that both AOM and *p*-CA improved cognitive functions, reduced anxiety, and increased hippocampal neurogenesis in the post-middle cerebral artery occlusion ischemic rats *in vivo* through activating BDNF/TrkB/AKT signaling pathway.

The study by Sasaki et al. investigated the neurodevelopmental and neuroprotective effects of the microalgae *Aurantiochytrium* sp. in a number of experimental models. They reported that *Aurantiochytrium* inhibited amyloid-β-induced cell death and increased ATP production in SH-SY5Y human neuroblastoma cells, increased proliferation of murine neurospheres, improved spatial learning and memory in the senescence-accelerated SAMP8 mice and enhanced neurogenesis in the mice hippocampal dentate gyrus.

The final article in this Research Topic by Achour et al. reported that natural flavone luteolin inhibited notch signaling and therefore inhibited the self-renewal of hNSCs, and directed the differentiation of hNSCs towards astrocytes likely via mediating WNT-β-catenin-BMP2-STAT3 pathways. *In vivo*, luteolin improved neuroinflammation by decreasing proinflammatory cytokine levels in mice astrocytes and sera and increased neurotransmitter and neurotrophic factor levels in the hypothalamus in lipopolysaccharide (LPS)-induced neuroinflammatory model of depression in mice. They have also presented a comprehensive whole-genome transcriptome analysis, which provided a detailed view of the changes in biological functions in mice hippocampus and brain-derived NSCs by luteolin administration and highlighted the possible therapeutic benefits of luteolin in neuroinflammatory and neurodegenerative diseases.

All these five papers had a common parameter-neurogenesis in hippocampus. Discovering adult hippocampal neurogenesis (AHN) led to a paradigm shift in neuroscience. Adult-born hippocampal neurons are one of the key mediators of hippocampus-dependent functions, such as learning, memory encoding, mood regulation, and stress response. Recent scientific evidence suggests that impairment of AHN underlies the pathophysiology of neurodegenerative and affective disorders (Mu and Gage, 2011; Baptista and Andrade, 2018; Toda et al., 2019; Gomes-Leal, 2021). Therefore, hippocampal neurogenesis-inspired therapies using plant-derived bioactive compounds offer a promising approach to reducing symptoms of neurodegenerative and mood disorders in humans (Zhang et al., 2014; Sasikumar et al., 2022).

In this article Research Topic, there are two interesting studies on perinatal stem cell human amniotic epithelial cells (hAECs/hAESCs) (Aonuma et al.; Uchida et al.). The hAECs are obtained from discarded term placenta and therefore are readily available. hAECs possess both ESC-like pluripotent potential and adult stem cell-like immunomodulatory properties. In recent years, stem-cell-based approaches using ESCs and iPSC have received great attention as effective drug screening tools. However, invasive extraction and expensive cell reprogramming and maintenance procedures as well as ethical constraints, limit the use of these types of stem cells for drug screening (Chen et al., 2014). In this context, hAECs offer a suitable alternative to ESCs and iPSCs (Miki et al., 2005; Murphy et al., 2010; Miki, 2018). In their study, Aonuma et al. considered hAECs as a drug screening tool and investigated the cardiac antifibrotic potential of a plant flavonol isorhamnetin in hAECs through whole-genome transcriptome analysis and then validated the findings in angiotensin II (AgII)-induced mice model of cardiac fibrosis and hypertrophy. On the other hand, Uchida et al. reported that isorhamnetin regulated early biological events to induce hepatic-lineage-specific differentiation in hAECs in the absence of any growth factor or cytokine. The differentiated hAECs expressed a subset of hepatic differentiation-related genes, induced cytochromes P450 (CYPs) mRNA levels, and showed some key functional properties of hepatic cells, including indocyanine green (ICG) uptake and release, glycogen storage, and urea secretion. There are few other studies that explored directed differentiation potential of different natural bioactive compounds in hAECs (Ferdousi et al., 2019; Bejaoui et al., 2021), while other studies explored improved therapeutic potential of hAECs in combinations with natural compounds (Ferdousi et al., 2020; Xu et al., 2021). These study findings would encourage multidirectional research approaches through integrating natural bioactive compounds with the existing hAECs research platforms (Ferdousi and Isoda, 2022).

The study by Kubo et al. has drawn much attention due to its promising findings on the hair growth potential of several polyphenols. Hair thinning and alopecia is more than just cosmetic concern and has a significant negative impact on the quality of life. To date, only two compounds, finasteride and minoxidil, are commonly used to improve hair loss conditions, but they are not without side effects. Therefore, there has been growing interest in natural bioactive compounds with properties that promote hair growth or limit hair loss as a safe alternative to drug-based therapy (Park and Lee, 2021). However, although many medicinal plants have been used anecdotally from ancient times to prevent hair loss, the scientific evidence is lacking about whether and how these plant-derived products are effective for the treatment of hair thinning and alopecia. In their study, Kubo et al. used an *in vitro* screening system in the human keratinocyte cell line, HaCaT, to identify polyphenols that can augment the expression of telomerase reverse transcriptase (TERT), a catalytic subunit of the enzyme telomerase, that activates the hair follicle bulge stem cells and triggers the initiation of new hair follicle growth phase and thereby promotes hair growth. They have identified the polyphenols-fisetin and resveratrol as potent hTERT-augmenting compounds that also enhanced β-catenin and hair growth cycle-related growth factors *in vitro* and *in vivo*.

Moqbel et al. investigated the anti-inflammatory effects of tectorigenin, an extracted component of *Belamcanda chinensis*, on TNFα-stimulated tendon-derived stem cells (TDSCs) and an animal model of tendinopathy. TDSCs are pluripotent stem cells that control tendon homeostasis and play a central role in tendon regeneration and healing and, therefore, are considered a potential cell-based therapy for tendon injuries (Wei and Lu, 2021). Moqbel et al. showed that tectorigenin inhibited TNFα-induced matrix-degradation, inflammation, apoptosis, senescence, and osteogenic differentiation of TDSCs in NF-κB/MAPK-dependent manner *in vitro*. Furthermore, tectorigenin ameliorated tendinopathy in a tendon transection rat model. This study highlights the potential of plant-derived compounds with strong anti-inflammatory effects in tendinopathy and other musculoskeletal disorders.

The study by Ganbold et al. explored the effect of a new amphiphilic squalene derivative (HH-Sq) in comparison to squalene (Sq) on the adipocyte differentiation of adipose-derived stem cells (ASCs) obtained from type 2 diabetic subject. ASCs are adult stem cells that can be differentiated into mesodermal cell lineages, including adipocytes, osteocytes, myocytes, and chondrocytes and have the potential to be used for cell therapy in the treatment of insulin resistance, obesity, and type 2 diabetes (Mazini et al., 2020). On the other hand, Sq is a polyunsaturated hydrocarbon found in deep-sea shark liver oil, numerous plant oils and algae (Spanova and Daum, 2011). Ganbold et al. demonstrated that the amphiphilic HH-Sq, synthesized by adding mono ethylene glycol moiety to Sq, showed improved metabolism of adipocytes, enhanced energy homeostasis and insulin sensitivity, and importantly, in contrast to Sq, HH-Sq prevented excessive lipogenesis in the presence of adipocyte differentiation. This finding emphasizes the enhanced therapeutic potentials of synthesized derivatives from a natural compound in cell-based therapies.

He B. et al. reported that fraxinellone (FRA), the bioactive component isolated from the D. dasycarpus plant, inhibited the proliferation and migration of human osteosarcoma cells HOS and MG63 in a dose-dependent manner. FRA simultaneously induced osteosarcoma cell apoptosis and increased autophagy flux *in vitro*. The authors further demonstrated the anticancer effects of FRA in the xenograft orthotopic mice model. They have proposed that the anticancer effects of FRA were achieved through autophagy flux. Targeting autophagy is recognized as a promising therapeutic strategy to overcome drug resistance and reduce metastasis in osteosarcoma (Liao et al., 2019). The study findings of He B. et al. would strengthen the idea of exploiting more plant natural compounds as potential novel antitumor therapeutics for osteosarcoma by targeting autophagy pathways.

Taken together, the current Research Topic provides multidirectional insights on plant-derived natural compounds-based research in stem cells.
